# Telehealth Use in Primary Care Pediatrics During and After the COVID-19 Pandemic

**DOI:** 10.1001/jamanetworkopen.2025.44421

**Published:** 2025-11-19

**Authors:** Chloe A. Teasdale, Madhura S. Rane, Geetha Gopalan, Joseph Nahmias, Michael L. Rinke

**Affiliations:** 1 Department of Epidemiology and Biostatistics, Graduate School of Public Health and Health Policy, City University of New York, New York, New York; 2 Institute for Implementation Science in Population Health, City University of New York Graduate School of Public Health and Health Policy, New York, New York; 3 Department of Epidemiology, Mailman School of Public Health, Columbia University, New York, New York; 4 Silberman School of Social Work, Hunter College, City University of New York, New York, New York; 5 Montefiore Health System, Bronx, New York; 6 Department of Pediatrics, Children’s Hospital of Montefiore and Albert Einstein College of Medicine, Bronx, New York

## Abstract

This cohort study compares rates of telehealth care among children during and after the COVID-19 pandemic in New York, New York.

## Introduction

Telehealth service use expanded rapidly during the COVID-19 pandemic.^1^ While some studies show high use of telehealth for pediatric care early in 2020, few have examined telehealth throughout the pandemic and after.^2,3,4^ We describe telehealth use in pediatric primary care in a large urban health system 2020 to 2023.

## Methods

This cohort study was approved by the Einstein/Montefiore institutional review board with a waiver of informed consent based on minimal risk. We analyzed data on all pediatric primary care (well and sick) visits at Montefiore Health System (MHS) pediatric primary care and family medicine office in Bronx, New York. Patients younger than 19 years with at least 1 primary care visit between March 1, 2020, and December 31, 2023, were included.

We report proportions of primary care pediatric visits coded as virtual, indicating telehealth visits conducted via virtual platform (excluding telephone). We also measured number and proportion of unique children with at least 1 telehealth visit in the COVID-19 (March 1 to August 30, 2020) and post–COVID-19 (March 1 to August 30, 2023) periods. We used 2-sided χ^2^ tests to identify demographic characteristics, including age, sex, race and ethnicity, insurance type, and neighborhood income (median income by zip code, based on 2020 US Census data) for children most likely to have used telehealth service within the 2 periods. Race and ethnicity were reported separately by parents or caregivers and combined for analyses as American Indian, Alaskan Native, or Pacific Islander; Asian; Hispanic; non-Hispanic Black; and non-Hispanic White. Statistical significance was set at *P* ≤ .05. Analyses were conducted using R software version 2025.9.1.401 from May to September 2025.

## Results

A total of 680 791 visits were attended by 117 999 patients at 20 primary care pediatric and family medicine offices between March 2020 and December 2023. As a proportion of all visits, telehealth visits peaked in April 2020 (70.5%) and by December 2023, were 2.0% of visits (Figure).

**Figure.**
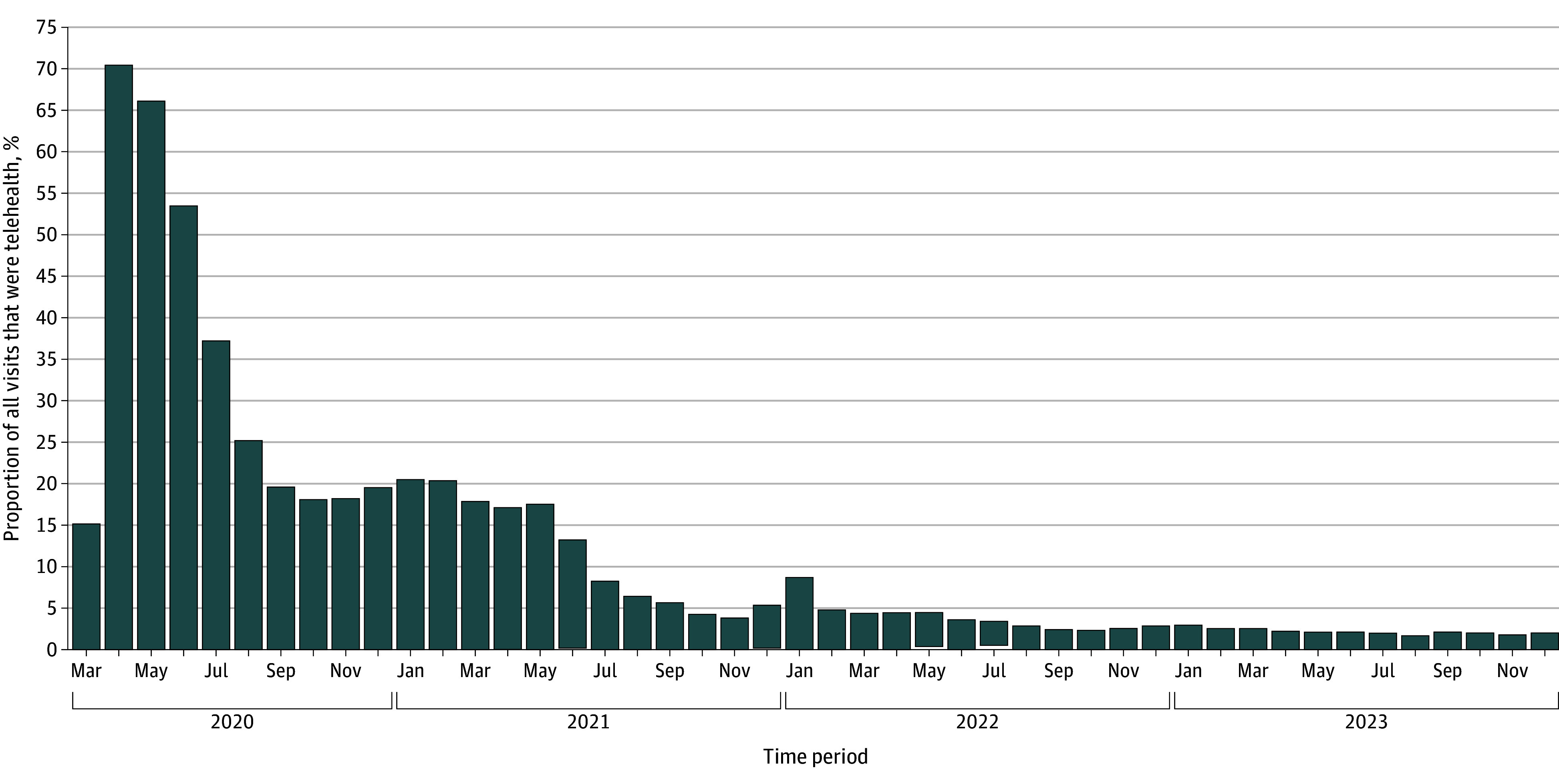
Proportion of All Pediatric Primary Care Visits via Telehealth Services Out of All Visits, March 2020 to August 2023, Bronx, New York In a linear regression model to estimate the change in proportion of visits by telehealth out of all pediatric primary visits from March 2020 to December 2023, the coefficient per months was −0.88 (95% CI, −1.12 to −0.64), representing approximately 1% decline per month and 11% decline per year during the period.

During the COVID-19 period, of 80 311 visits attended by 47 114 unique children, 27 853 (34.7%) were telehealth, and 19 400 children (41.2%) had 1 or more telehealth visits (Table). Almost half (46.0%) of adolescents aged 15 to 18 years attended a telehealth visit, compared with 39.7% of children aged 0 to 4 years (*P* < .001). Hispanic children were most likely to have telehealth visits (44.0%) compared with Asian (41.6%), non-Hispanic Black (40.5%), or non-Hispanic White (39.5%) children (*P* < .001). Children without insurance (51.0%) were most likely to have telehealth visits compared with children with public (41.0%) or private (32.1%) insurance (*P* < .001).

**Table.** Telehealth Visits and Proportions of Children and Adolescents (0-18 Years) With ≥1 Telehealth Visit in the COVID-19 Period (March-August 2020) and Post–COVID-19 Period (March-August 2023)

Characteristic	COVID-19 period (March-August 2020)					Post–COVID-19 period (March-August 2023)				
	Total visits, No.	Telehealth visits, No. (row %)	Unique children, No.	Children with ≥1 telehealth visit, No. (row %)	*P* value^a^	Total visits, No.	Telehealth visits, No. (row %)	Unique children, No.	Children with ≥1 telehealth visit, No. (row %)	*P* value^a^
No. (%)	80 311	27 853 (34.7)	47 114	19 400 (41.2)		93 078	1807 (1.9)	53 949	1504 (2.8)	
Age, y^b^										
0-4	35 282 (43.9)	10 316 (29.2)	16 599	6584 (39.7)	<.001	38 399 (41.3)	661 (1.7)	16 679	553 (3.3)	<.001
5-9	16 918 (21.1)	6538 (38.7)	11 559	4845 (41.9)		21 777 (23.4)	463 (2.1)	14 123	377 (2.7)	
10-14	16 272 (20.3)	5878 (36.1)	11 310	4437 (39.2)		19 038 (20.5)	361 (1.9)	13 638	302 (2.2)	
15-18	11 839 (14.7)	5121 (43.3)	7676	3534 (46.0)		13 864 (14.9)	322 (2.3)	9509	272 (2.9)	
Sex										
Male	40 094 (49.9)	14 369 (35.8)	23 619	9504 (40.2)	<.001	47 261 (50.8)	985 (2.1)	27 361	795 (2.9)	.09
Female	40 214 (50.1)	13 483 (33.5)	23 523	9895 (42.1)		45 808 (49.2)	822 (1.8)	26 583	709 (2.7)	
Race and ethnicity^c^										
American Indian, Alaskan Native, or Pacific Islander	891 (1.1)	246 (27.6)	455	183 (40.2)	<.001	958 (1.0)	10 (1.0)	565	10 (1.8)	<.001
Asian	3223 (4.0)	1107 (34.4)	1660	690 (41.6)		4429 (4.8)	49 (1.1)	2149	41 (1.9)	
Black, non-Hispanic	20 747 (25.8)	7240 (34.9)	13 161	5327 (40.5)		23 282 (25.0)	392 (1.7)	14 762	332 (2.3)	
Hispanic	32 571 (40.6)	12 077 (37.1)	18724	8229 (44.0)		38579 (41.5)	829 (2.2)	22 010	674 (3.1)	
White, non-Hispanic	4362 (5.4)	1482 (34.0)	2460	972 (39.5)		4939 (5.3)	164 (3.3)	2484	127 (5.1)	
Other	8047 (10.0)	2692 (33.5)	4734	1861 (39.3)		9554 (10.3)	173 (1.8)	5399	146 (2.7)	
Missing or not disclosed	10 470 (13.0)	3009 (28.7)	5950	2138 (35.9)		11 337 (12.2)	190 (1.7)	6580	174 (2.6)	
Insurance										
Public^d^	77 235 (96.2)	26 823 (34.7)	45 505	18 665 (41.0)	<.001	90 340 (97.1)	1726 (1.9)	52 478	1432 (2.7)	<.001
Private	1890 (2.4)	457 (24.2)	1057	339 (32.1)		2075 (2.2)	46 (2.2)	1150	40 (3.5)	
Self-pay	1186 (1.5)	573 (48.3)	1013	517 (51.0)		663 (0.7)	35 (5.3)	568	35 (6.2)	
Neighborhood income^e^										
>Median	40 066 (49.9)	13 585 (33.9)	23 480	9485 (40.4)	<.001	47 111 (50.6)	1067 (2.3)	26 775	872 (3.3)	<.001
<Median	37 737 (47.0)	13 404 (35.5)	22 214	9347 (42.1)		44 645 (48.0)	705 (1.6)	26 297	601 (2.3)	
Missing	2508 (3.1)	864 (34.5)	1450	568 (39.2)		1322 (1.4)	35 (2.7)	877	31 (3.5)	

^a^ *P* values from χ^2^ test comparing expected vs observed values for variables for unique children within each time period (to identify children most likely to have received telehealth services in each period).

^b^ Age of patients on March 1, 2020 (COVID-19 period), and March 1, 2023 (post–COVID-19 period).

^c^ Race and ethnicity data were collected separately as reported by parents or caregivers during medical visits. For analyses, we combined race and ethnicity into 1 variable; any child indicated to be Hispanic was classified as Hispanic; all other children were classified according to the race indicated by parents or caregivers.

^d^ Public insurance includes Medicare and Children Health Insurance Program.

^e^ Neighborhood income was estimated based on median household income in the 2020 US Census using children’s postal zip code (billing/residence). For the analyses, participants were described as having higher income' if their neighborhood median income was above the overall median income of $26 160 across zip codes included in the analyses, and lower income if it was below the median.

In the post–COVID-19 period, of 93 078 visits with 53 949 unique children, 1807 (1.9%) were via telehealth, and 2.8% of all children had 1 or more telehealth visits (Table). In the post–COVID-19 period, children who were non-Hispanic White were most likely to have telehealth visits (5.1%) compared with those who were Asian (1.9%), Hispanic (3.1%), or non-Hispanic Black (2.3%) (*P* < .001). Children without health insurance were most likely to have telehealth visits (6.2%) compared with those with public (2.7%) or private (3.5%) insurance (*P* < .001) (Table).

## Discussion

Findings from this large cohort study of children and adolescents in New York, New York provide further evidence of the rapid integration of telehealth into pediatric primary care early in the COVID-19 pandemic. A strength of our study is large sample size and examination of 45 months of data. Limitations include that our cohort included publicly insured children living in urban areas, which may limit generalizability to urban centers.

Our results indicate Hispanic children and those from low-income neighborhoods were able to access telehealth services during the peak of the pandemic, despite equity concerns.^5,6^ Postpandemic, we observed a return to almost all in-person care, as well as greater use of telehealth among White children and those from higher-income neighborhoods. We cannot determine whether these changes are a result of evolving preferences or lack of access. Further research is needed to understand clinician, patient, and caregiver attitudes toward telehealth, as is research identifying any impact of telehealth on health outcomes in children and adolescents.

## Notes

Supplement.
Data Sharing Statement
