# Supplementary material for: Telehealth Use in Primary Care Pediatrics During and After the COVID-19 Pandemic
Source: JAMA Netw Open. 2025 Nov 19;8(11):e2544421. doi: 10.1001/jamanetworkopen.2025.44421 (PMC12631480; doi:10.1001/jamanetworkopen.2025.44421)
Supplement: Supplement. — Data Sharing Statement [file jamanetwopen-e2544421-s001.pdf]

Data Sharing Statement

Teasdale. Telehealth Use in Primary Care Pediatrics During and After the COVID-19 Pandemic. *JAMA Netw Open*. Published November 19, 2025.
doi:10.1001/jamanetworkopen.2025.44421

Data
Data available: No

Additional Information
Explanation for why data not available: The data came from medical records including dates of service so these data cannot be made available.
